# Examining the Influence of Housing Conditions and Daily Greenspace Exposure on People’s Perceived COVID-19 Risk and Distress

**DOI:** 10.3390/ijerph19148876

**Published:** 2022-07-21

**Authors:** Jianwei Huang, Mei-Po Kwan

**Affiliations:** 1 Institute of Space and Earth Information Science, The Chinese University of Hong Kong, Shatin, Hong Kong, China; jianwei.huang@link.cuhk.edu.hk; 2 Department of Geography and Resource Management, The Chinese University of Hong Kong, Shatin, Hong Kong, China

**Keywords:** perceived COVID-19 risk, distress, housing conditions, greenspace exposure, daily activity

## Abstract

Many people have worried about COVID-19 infection, job loss, income reduction, and family conflict during the COVID-19 pandemic. Some social groups may be particularly vulnerable due to their residential neighborhoods and daily activities. On the other hand, people’s daily exposure to greenspace offers promising pathways for reducing these worries associated with COVID-19. Using data collected with a questionnaire and a two-day activity diary from two typical neighborhoods in Hong Kong, this study examines how people’s housing conditions and daily greenspace exposure affect their perceived COVID-19 risk and distress (i.e., worries about job loss, income reduction, and family conflict) during the pandemic. First, the study compares people’s perceived COVID-19 risk and distress based on their residential neighborhoods. Further, it examines the associations between people’s perceived COVID-19 risk and distress with their housing conditions and daily greenspace exposure using ordinal logistic regression models. The results indicate that living in a high-risk neighborhood, being married, renting a residential unit, and living in a large household are significantly associated with a higher neighborhood-based perceived COVID-19 risk and distress during the pandemic. In addition, people also reported lower mobility-based perceived COVID-19 risk when compared to their neighborhood-based perceived COVID-19 risk, while they still have a high perceived COVID-19 risk in their occupational venues if they have to work in a high-risk district (e.g., Kowloon). Lastly, daily greenspace exposure (i.e., woodland) could reduce people’s perceived COVID-19 risk and distress. These results have important implications for the public health authority when formulating the measures during the COVID-19 pandemic.

## 1. Introduction

The COVID-19 pandemic is one of the most serious public health crises that emerged since January 2020. In the early stage, certain non-pharmaceutical interventions (e.g., stay-at-home order, border control, and closure of public facilities) are considered effective in mitigating the spread of COVID-19. Since these control measures can reduce people’s face-to-face contact, they can help reduce the transmission of COVID-19 [1,2,3]. However, COVID-19 and the control measures have various impacts on different social groups because of different sociodemographic and environmental determinants. The effectiveness and potential benefits of using non-pharmaceutical interventions highly depend on the cooperation of the general population and the improvement of personal protection, which could be affected by people’s perceived risk during the pandemic [4,5].

Along this line, previous studies have found that certain social groups have a higher perceived COVID-19 risk than others in different countries. For example, using data collected in four major cities in the U.S. (i.e., Seattle, Los Angeles, Chicago, and New York City) through online surveys, Qin et al. [6] found that people with more conservative political views have lower levels of personal perceived COVID-19 risk than those with more liberal political views. Yıldırım et al. [7] also found that females reported higher perceived COVID-19 risk than males based on a dataset of 4536 Turkish collected through an online survey. However, by using data through an online survey in the U.S., Alschuler et al. [8] reported that gender is not associated with people’s perceived COVID-19 risk, while age plays an important role in people’s perceived COVID-19 risk. One of the reasons for these different findings may be due to the use of online surveys, which may have some sampling bias [9,10].

Besides, COVID-19 mitigation measures also have enormous economic impacts, including financial hardship and job loss, which may worsen people’s distress. Specifically, many people (e.g., people working in the service sector) have to face precarious employment and low income due to the closures of their workplaces caused by the restriction measures, an infected co-worker, or loss of business [11,12]. Governments of different countries (e.g., Germany, Spain, the U.K., Italy, Switzerland, the U.S., and Thailand) have reported that the unemployment rate would be high because of the pandemic [13,14,15,16,17]. Meanwhile, family conflicts among household members may increase due to the restriction measures (e.g., stay-at-home orders) because people spend more time with their family members. Studies have observed unprecedented numbers of divorce and domestic violence cases in China, South Africa, India, France, and Australia after the lockdown [18]. Thus, people’s worries about job loss, income reduction, and family conflict due to the COVID-19 pandemic may lead to greater anxiety symptoms [19].

Although previous studies have paid significant attention to examining people’s perceived COVID-19 risk and distress during the pandemic, they did not examine whether people’s perceived COVID-19 risk and distress may be different due to their residential neighborhoods and daily mobility. Specifically, different neighborhoods have various socio-demographic characteristics (e.g., population density, age, and gender) and built-environment features (e.g., green space, building density, and transport accessibility), which would shape people’s daily mobility and thus may further render some neighborhoods riskier than others [2,20]. Thus, people’s perceived COVID-19 risk might be different due to their various residential neighborhoods. The uneven spatial distribution of COVID-19 transmission may lead some social groups to be doubly disadvantaged if they live in high-risk neighborhoods. Moreover, people’s perceived COVID-19 risk may be different if their daily mobility is considered. Since most people travel to different venues outside of their residential neighborhoods in their daily life, they are exposed to different levels of COVID-19 risk [21]. Hence, people’s distress may dramatically increase because of the fear of exposure to COVID-19 and the enormous economic impacts of the pandemic. Thus, it is critical to investigate people’s perceived COVID-19 risk and distress in different neighborhoods in order to effectively identify the vulnerable social groups during the pandemic.

In addition, people’s perceived COVID-19 risk and distress may also be affected by their housing conditions and greenspace exposure associated with their daily activities. For instance, by using data from 3135 counties in the U.S. from a public source, Ahmad et al. [22] found that people with poor housing conditions (e.g., overcrowded and high housing costs) have high COVID-19 incidence and mortality rate. Wang et al. [23] further revealed that there are multiple transmission routes (e.g., spreading of stack aerosols with the virus via faulty drainage pipes) and a high transmission rate in buildings with poor housing conditions (e.g., poor ventilation) in Hong Kong. Meanwhile, using the Instagram posts dataset collected from four high-density Asian cities (i.e., Hong Kong, Singapore, Tokyo, and Seoul), Lu et al. [24] found that the usage of greenspace increased in the cities during the COVID-19 pandemic. Ma et al. [25] further concluded that the increased number of visitors to greenspace (i.e., country parks) in Hong Kong may be due to the implementation of appropriate visitor management measures (e.g., social distancing in country parks), which could make the perceived benefits of visiting country parks outweigh the perceived COVID-19 risks. In addition, Johnson et al. [26] also indicated that COVID-19 transmission risk was significantly lower in areas with more greenspace (i.e., open and recreational space). Slater et al. [27] recommended keeping greenspace (i.e., parks, recreational open spaces, and country parks) open and accessible during the pandemic, which has positive benefits on people’s physical and mental health. By using online survey data from Portugal and Spain, Ribeiro et al. [28] also found that people exposed to greenspace reported better psychological health outcomes than those who did not go outside during the pandemic. The possible causal pathways about the benefits of greenspace on people’s psychological well-being have been proposed: greenspace can reduce people’s exposure to environmental stressors while restoring attention and facilitating physical activity or social cohesion [29,30].

Therefore, people might have different levels of perceived COVID-19 risk and distress according to their housing conditions and daily exposure to greenspace, although they may live in the same residential neighborhoods. It is thus important to examine the associations between people’s perceived COVID-19 risk and distress with their housing conditions and daily greenspace exposure. The new knowledge generated can assist public health authorities in enhancing the effectiveness of supporting measures by targeting specific vulnerable social groups during the pandemic. On the other hand, certain housing conditions and greenspace can be identified and dynamically managed to reduce COVID-19 transmission risk factors while maintaining people’s psychological well-being.

This study thus seeks to bridge the abovementioned gaps by examining how people’s housing conditions and daily greenspace exposure may influence their perceived COVID-19 risk and distress in a high-density city. Using data collected with a questionnaire and a two-day activity diary in two typical neighborhoods (i.e., Sham Shui Po and Tin Shui Wai) in Hong Kong, we first compare people’s perceived COVID-19 risk and distress (i.e., worries about job loss, income reduction, and family conflict) based on their residential neighborhoods. Then, the associations between people’s perceived COVID-19 risk and distress with housing conditions and daily greenspace exposure are examined by using ordinal logistic regression models. Finally, we discuss how the findings reflect the vulnerable social groups in Hong Kong and their implications for public health during the COVID-19 pandemic.

## 2. Dataset and Methodology

### 2.1. Study Areas

**Figure 1:**
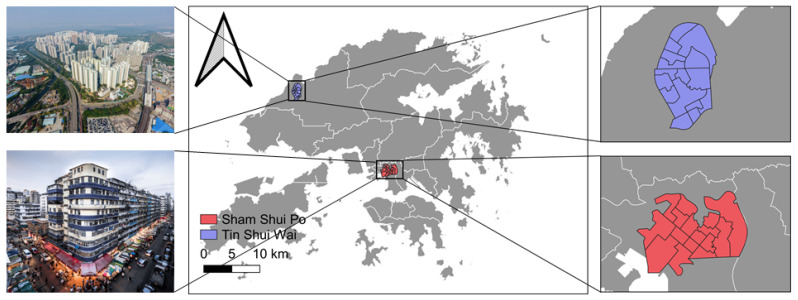
Study area.

This study focuses on the Sham Shui Po and Tin Shui Wai neighborhoods in Hong Kong (Figure 1). We chose these two neighborhoods because they represent two typical neighborhoods in Hong Kong during the pandemic. Sham Shui Po is in the center of the city within easy reach by Hong Kong’s efficient transport system. It is an old urban area developed in the 1920s with an average population density of 43,381 per km^2^ in 2016. Tin Shui Wai is in the suburban area of Hong Kong. It is a comprehensively planned new town built in the 1980s, with an average population density of 66,565 per km^2^ in 2016 [31]. The sociodemographic characteristics and built-environment features also differ between the two neighborhoods. Sham Shui Po is regarded as one of the poorest neighborhoods in Hong Kong. Specifically, it has many old buildings (or tenements) and considerable substandard housing with limited maintenance (e.g., faulty piping and poor ventilation). The neighborhood has vibrant local economic activities since it is also one of the most diverse (e.g., diverse races, nationalities, restaurants, and so on) and densely packed neighborhoods [32]. Dissimilar from Sham Shui Po, Tin Shui Wai is a well-planed new town, which mainly consists of public housing and middle-income private housing with much better sociodemographic and built environment characteristics.

Hong Kong implemented a strict zero-COVID strategy in January 2020. Specifically, the strict restriction measures included border control (e.g., in-bound travelers were required to take a 14-day self-quarantine), social distancing (e.g., suspension of public facilities, schools, closures of bars, and clubs), contact tracing and location disclosure (e.g., in-depth interviews, and disclosure of buildings and venues visited by infected people in the 14 days before their infection was confirmed) [9]. It should be noted that Hong Kong had suffered four different waves of COVID-19 outbreaks from January 2020 to May 2021, which reported less than 15,000 total confirmed cases. In addition, Sham Shui Po has been a high-risk neighborhood during the pandemic because it has suffered repeated COVID-19 outbreaks from January 2020 to May 2021 [33,34,35]. Meanwhile, Tin Shui Wai was a low-risk neighborhood for COVID-19 between January 2020 and May 2021.

### 2.2. Data Collection

**Figure 2:**
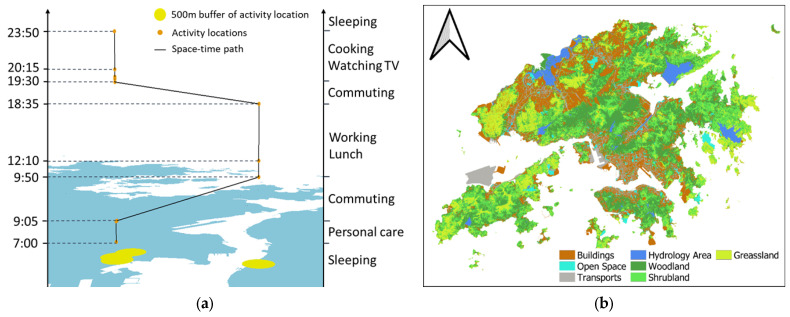
Activity diary data and land use dataset: (**a**) The activity diary illustrated by the example of one participant’s workday; (**b**) Land-use dataset.

A survey was conducted in the two neighborhoods from April 2021 to September 2021. Using a stratified sampling approach, we recruited a total of 221 participants aged between 18 and 65 years old to participate in the survey. Specifically, we first invited the participants to attend a face-to-face briefing session to introduce the survey. Then, each participant was asked to complete a questionnaire about their socio-demographic attributes, housing conditions, perceived COVID-19 risk, and distress during the briefing session. Third, the participants were further asked to complete a two-day activity diary (i.e., one weekday and one weekend day) (Figure 2a) in the following days. Hence, the survey collected information about participants’ sociodemographic attributes, housing conditions, daily activity locations (i.e., places and venues visited and activity duration), perceived COVID-19 risk, and distress during the pandemic. It should be noted that although global positioning systems (GPS) can offer the possibility of collecting high-resolution spatiotemporal data, the satellite signals are very often obstructed by tall buildings or large structures in Hong Kong since the city has a built environment characterized by compactivity and density. Thus, GPS may provide an insufficient number of Global Navigation Satellite System (GNSS) measurement data for successful position determination in the city [36,37]. Hence, we apply an activity diary method to obtain accurate activity positions and times from the participants.

Finally, the survey obtained valid data from 219 participants (107 in Sham Shui Po, and 112 in Tin Shui Wai), and the samples of the two neighborhoods represent their respective populations well. Table 1 presents the participants’ sociodemographic attributes in the two communities. The proportion of students in the Sham Shui Po sample is lower than the proportion of students in the neighborhood based on census data, while it is higher for the Tin Shui Wai sample. In addition, the percentages of the low-income group (less than HKD 20,000) and older adults (45–64 years old) are lower than that in the census-based profiles of the two neighborhoods. The composition of other demographic characteristics such as gender ratio and employment status are generally similar to the census-based profiles of the two neighborhoods. Note that there were barely any new COVID-19 confirmed cases among communities in Hong Kong from April 2021 to September 2021. The survey protocol and questionnaire were reviewed and approved by the Institutional Review Boards (IRBs) of the authors’ university.

The sociodemographic attributes of the participants collected via the survey include their gender, age, monthly household income, educational attainment, employment status, working place, and marital status. The housing conditions include participants’ house type (e.g., public housing, private housing, *tong lau* [old tenement buildings], and subdivided flats/units), household size (i.e., the number of household members), monthly household rent or mortgage payment, and homeownership (i.e., rented or owned). The survey questionnaire also collected participants’ perceived COVID-19 risk and distress during the pandemic in Hong Kong. The questions about people’s perceived COVID-19 risk and distress are listed in Table 2. Specifically, the perceived COVID-19 risk includes two response items: (1) participants’ perceived COVID-19 risk in their residential neighborhood during the pandemic (i.e., the neighborhood-based perceived COVID-19 risk); (2) participants’ perceived COVID-19 risk in their daily activity venues during the pandemic (i.e., the mobility-based perceived COVID-19 risk). Both neighborhood-based and mobility-based perceived COVID-19 risk of the participants is recorded based on a six-point scale (from 1 to 6). For example, “1” indicates “zero COVID-19 confirmed cases”, while “6” indicates “the transmission of COVID-19 is severe”. Questions concerning participants’ distress include three items: (1) participants’ worry about job loss during the pandemic; (2) participants’ worry about income reduction during the pandemic; (3) participants’ worry about family conflict during the pandemic. The three response items of distress are also recorded based on a six-point scale (from 1 to 6). For instance, “1” indicates “never”, while “6” indicates “always”. To measure participants’ daily greenspace exposure, we also collect a land-use dataset (Figure 2b), which is provided by the Hong Kong Planning Department in 2018. The dataset includes 4 different types of greenspace with a spatial resolution of 10 m × 10 m: (1) open space and recreational land, which include parks, stadiums, playgrounds, and recreational facilities; (2) grassland; (3) shrubland; and (4) woodland.

### 2.3. The Associations between People’s Perceived COVID-19 Risk and Distress with Their Housing Environment Conditions and Greenspace Exposure

This subsection focuses on examining the associations between people’s perceived COVID-19 risk and distress with their housing conditions and daily greenspace exposure by using regression models. Socio-demographic features are also considered in the models. Table 3 presents the socio-demographic features, housing conditions, and greenspace exposure we derived based on the dataset described in Section 2.2 above. Specifically, the socio-demographic features include participants’ residential neighborhoods (Sham Shui Po: 1; Tin Shui Wai: 0), gender (female: 1; male: 0), age group 1 (18–24 years old: 1; otherwise: 0), age group 2 (45+ years old: 1; otherwise: 0), higher education (yes: 1; no: 0), marital status (married: 1; single, widowed, or divorced: 0), working place 1 (Hong Kong island: 1; otherwise: 0), working place 2 (Kowloon: 1; otherwise: 0), monthly household income 1 (<HKD 20,000: 1; otherwise: 0), monthly household income 2 (>HKD 40,000: 1; otherwise: 0), student (yes: 1; no: 0), full-time employed (yes: 1; no: 0), and housewife (yes: 1; no: 0). The housing conditions include house ownership (rented: 1; owned: 0), household size (i.e., the number of household members in participants’ residential units), house type 1 (public house: 1; otherwise: 0), house type 2 (*tong lau* or subdivided units: 1; otherwise: 0), monthly household rent/loan 1 (HKD 1–4000: 1; otherwise: 0), monthly household rent/mortgage payment 2 (HKD 4000–10,000: 1; otherwise: 0), and monthly household rent/mortgage payment 3 (>HKD 10,000: 1; otherwise: 0).

The daily greenspace exposure of participants is measured according to their residential locations and daily activity diaries. Specifically, it includes two different measurements: (1) neighborhood-based greenspace exposure, and (2) activity-based greenspace exposure. The neighborhood-based greenspace exposure includes open space and recreational land, woodland, shrubland, and grassland, which are assessed by the area of each type of the greenspace land inside a buffer area of 500 m (i.e., walking distance < 10 min) around participants’ home locations [38,39]. The mobility-based greenspace exposure is assessed by the sum of the time-weighted area of each type of greenspace land inside a buffer area of 500 m around participants’ daily activity locations, as Equation (1) shows:

(1) $$GE_{type}=\left(\frac{S_{v1}}{S_{b500}}\times \frac{t_{1}}{48}+\frac{S_{v2}}{S_{b500}}\times \frac{t_{2}}{48}+\ldots +\frac{S_{vn}}{S_{b500}}\times \frac{t_{n}}{48}\right)$$

where $GE_{type}$ measures participants’ exposure to a certain type of greenspace (i.e., open space and recreational land, woodland, shrubland, or grassland) in their daily activities; $S_{b500}$ is the area of 500 m buffer; $S_{vn}$ is the area of greenspace land coverage in the nth activity location buffer, and so on; $t_{n}$ is the duration the participant spent in the nth activity space, and so on.

We estimate five ordinal logistic regression models to examine the association between the selected features with participants’ perceived COVID-19 risk and distress. The dependent variable of Model 1 is participants’ neighborhood-based perceived COVID-19 risk, while the independent variables of Model 1 are the selected socio-demographic features, housing conditions, and neighborhood-based greenspace exposure. The dependent variables of Models 2–5 are participants’ mobility-based perceived COVID-19 risk (Model 2), worries about job loss (Model 3), income reduction (Model 4), and family conflict (Model 5). The independent variables of Models 2–5 are the selected socio-demographic features, housing conditions, and mobility-based greenspace exposure. The variance inflation factor (VIF) is used to test the multicollinearity of the variables before estimating the regression models. The results show that there is no significant collinearity among the independent variables (i.e., VIF < 5).

## 3. Results

### 3.1. Statistical Description of People’s Perceived COVID-19 Risk and Distress during the Pandemic

In this subsection, we examine people’s perceived COVID-19 risk and their distress during the pandemic in the two neighborhoods (i.e., Sham Shui Po and Tin Shui Wai). Table 4 shows the results of the descriptive statistics, which include the mean values and standard deviation values of people’s neighborhood-based and mobility-based perceived COVID-19 risk and their worries about job loss, income reduction, and family conflict during the pandemic. In addition, we also use paired sample t-test to assess the differences between people’s neighborhood-based and mobility-based perceived COVID-19 risk in the two neighborhoods. Table 5 presents the differences in people’s perceived COVID-19 risk and distress during the pandemic in the two neighborhoods using Mann-Whitney U test, which is a nonparametric equivalent of the paired-sample t-test. The method does not assume the data to follow normal distributions and thus it can be used when this assumption is violated [40]. Table 6 focuses on the rate of people’s high perceived COVID-19 risk and severe distress in the two neighborhoods during the pandemic. The rates of high perceived COVID-19 risk and severe distress indicate the percentage of participants who selected 4, 5, and 6 for the questions about their perceived COVID-19 risk and distress.

First, we find that people’s neighborhood-based perceived COVID-19 risk, worries about job loss, income reduction, and family conflict in the two neighborhoods are significantly different. Specifically, people who live in Sham Shui Po have both higher neighborhood-based perceived COVID-19 risk and distress than those who live in Tin Shui Wai. In addition, Sham Shui Po has higher rates of people who reported high neighborhood-based perceived COVID-19 risk (43%), severe worries about job loss (53%), income reduction (59%), and family conflict (45%) than those who live in Tin Shui Wai, which has 19% people reporting high neighborhood-based perceived COVID-19 risk, 29% people reported severe worry about job loss, and 38% reported severe worries about income reduction and family conflict. One possible reason is that Sham Shui Po is a neighborhood with many old buildings, which are potential hotbeds of COVID-19 transmission [41]. Specifically, the neighborhood had suffered repeated COVID-19 outbreaks from January 2020 to May 2021 [33,34,35]. Therefore, the government implemented strict restriction measures (i.e., lockdown of several block areas) in the neighborhood when a COVID-19 outbreak happened. The social isolation that resulted from the restriction measures, coupled with the fear of the risk of exposure to COVID-19 in the dilapidated housing conditions, may curtail their routine daily activities and social interactions. All of these may lead to an increase in their neighborhood-based perceived COVID-19 risk and distress [42].

Second, the results also indicate that people who live in the two neighborhoods have similar mobility-based perceived COVID-19 risk patterns, which are significantly different from people’s neighborhood-based perceived COVID-19 risk patterns. Specifically, people’s mobility-based perceived COVID-19 risk (i.e., the mean value is 2.50 for participants from Sham Shui Po, and 2.55 for participants from Tin Shui Wai) is lower than people’s neighborhood-based perceived COVID-19 risk (i.e., the mean value is 3.40 for participants in Sham Shui Po and 2.95 for participants in Tin Shui Wai). The results imply that ignoring people’s daily mobility might overestimate their perceived COVID-19 risk. The potential reasons include that people have to follow social distancing regulations implemented by the Hong Kong Government when they are conducting their daily activities. Moreover, people may avoid going to the high-risk places after obtaining such information via the televised daily briefings from the health authority, which discloses the venues and buildings visited by the confirmed cases in the past 14 days.

### 3.2. The Associations between People’s Perceived COVID-19 Risk with Their Housing Conditions and Daily Greenspace Exposure

In this subsection, we focus on examining the associations between people’s perceived COVID-19 risk with their housing conditions and daily greenspace exposure using ordinal logistic regression (Models 1–2). The dependent variables of the regression models are people’s neighborhood-based perceived COVID-19 risk (Model 1), and people’s mobility-based perceived COVID-19 risk (Model 2). The independent variables are people’s social-demographic features, housing conditions, and neighborhood-based greenspace exposure in Model 1. Meanwhile, the independent variables are people’s social-demographic features, housing conditions, and mobility-based greenspace exposure in Model 2.

Table 7 presents the results of Models 1–2. First, we find that living in Sham Shui Po has a significant positive association with people’s neighborhood-based perceived COVID-19 risk, while it does not have a significant association with people’s mobility-based perceived COVID-19 risk. This is in line with the findings in Section 3.1 above. Meanwhile, the results indicate that people working in Kowloon (compared to people working in other districts) have higher mobility-based perceived COVID-19 risk. One of the potential reasons is that Kowloon is a district that has experienced COVID-19 outbreaks between January 2020 and May 2021 [35]. In addition, these results also imply that people have a high perceived COVID-19 risk in occupational venues because of the potential transmissions in their workplaces [43,44].

Second, the results demonstrate that having a high educational degree has a significant positive association with people’s neighborhood-based and mobility-based perceived COVID-19 risk. Furthermore, married people (compared to people who are single, widowed, or divorced) have higher neighborhood-based perceived COVID-19 risk, while people with a high monthly household income (>HKD 40,000, compared to the middle and low monthly household income group) have a lower neighborhood-based perceived COVID-19 risk. The results are consistent with the findings of previous studies, which found that educational status, income level, and marital status have strong associations with people’s perceived COVID-19 risk [45,46,47,48].

Third, the results show that renting a residential unit and having a low monthly rental or mortgage payment (HKD 1000–4000) have significant positive associations with people’s neighborhood-based perceived COVID-19 risk. It should be noted that people who pay a low monthly rent (HKD 1000–4000) can only afford a tiny unit with poor housing conditions in Hong Kong. These tiny units usually have limited maintenance (e.g., faulty piping and poor ventilation), which may increase people’s neighborhood-based perceived COVID-19 risk.

Lastly, the results also indicate that people’s daily exposure to woodland and shrubland has significant negative associations with their mobility-based perceived COVID-19 risk. In other words, the result implies that higher exposure to greenspace (woodland and shrubland) could reduce people’s perceived COVID-19 risk associated with their daily activities.

### 3.3. The Associations between People’s Distress with Their Housing Conditions and Daily Greenspace Exposure

In this subsection, we further examine the associations between people’s distress with their housing conditions and daily greenspace exposure using ordinal logistic regression analysis (Models 3–5). The dependent variables of the regression models are people’s worry about job loss (Model 3), people’s worry about income reduction (Model 4), and people’s worry about family conflict (Model 5). The independent variables are people’s socio-demographic features, housing conditions, and mobility-based daily greenspace exposure in Models 3–5.

Table 8 shows the results of Models 3–5. First, we find that living in Sham Shui Po (compared to living in Tin Shui Wai) has significant positive associations with people’s worries about job loss, income reduction, and family conflict. This is in line with the findings reported in Section 3.1 above. Meanwhile, the results suggest that females (compared to males) have a higher level of worry about job loss. The result is consistent with previous studies, which indicated that women are more likely to lose their jobs permanently than men because of the COVID-19 pandemic [49]. Furthermore, the results indicate that young people (compared to people who are older than 25) have a higher level of worry about family conflict. This result is similar to previous studies, which reported that young people have a serious worry about conflicts with their family members (e.g., parents) during the pandemic because of the stay-at-home orders and closure of schools [50,51].

Second, the results suggest that married people (compared to people who are single, widowed, or divorced) and people with a large household size (i.e., a high number of family members in the same household) have severe worries about job loss, income reduction, and family conflict. The results imply that people who have a big family (i.e., married and have a large household size) may have a double burden on their household financial status and family conflicts during the pandemic. It should be noted that previous studies have also observed this phenomenon in China [52], the U.S. [53,54], Austria [55], and Italy [56]. Meanwhile, we find that people who rent a residential unit (compared to people who own a residential unit or house) have severe worries about job loss and income reduction. The result also implies that people who live in a rented unit may have limited financial capacity to support themselves through the COVID-19 pandemic [57,58].

Lastly, the results show that people’s daily exposure to woodland could significantly decrease their worries about job loss, income reduction, and family conflict. These findings support previous findings, which reported consistent associations between people’s daily greenspace exposure and lower levels of distress [59,60,61].

## 4. Discussion

This study seeks to examine the associations between people’s perceived COVID-19 risk and distress with their housing conditions and daily greenspace exposure in a high-density city. Specifically, people’s perceived COVID-19 risk includes their neighborhood-based and mobility-based perceived COVID-19 risk, while people’s distress includes their worries about job loss, income reduction, and family conflict. By analyzing data collected with a questionnaire and a two-day activity diary from two typical neighborhoods (i.e., Sham Shui Po and Tin Shui Wai) in Hong Kong, the study first compares people’s neighborhood-based and mobility-based perceived COVID-19 risk between the two neighborhoods as well as their worries about jobs loss, income reduction, and family conflict. Then, we examine the associations between people’s perceived COVID-19 risk and distress with their housing conditions and daily greenspace exposure using ordinal logistic regression models. The main findings of the study are summarized as follows.

Our findings first reveal that people’s residential neighborhoods, housing conditions, and daily greenspace exposure play important roles in their perceived COVID-19 risk and distress during the pandemic. Specifically, people who live in Sham Shui Po (compared to people who live in Tin Shui Wai) have higher perceived neighborhood-based COVID-19 risk and severe worries about job loss, income reduction, and family conflict. The results support conclusions from previous studies, which indicated that people may be doubly disadvantaged if they live in a residential neighborhood with a high risk of COVID-19 transmission [35,62,63].

Second, we also find that people’s mobility-based perceived COVID-19 risk is significantly lower than people’s neighborhood-based perceived COVID-19 risk, while they still have a higher perceived COVID-19 risk in their occupational venues if they have to work in a high-risk district (e.g., Kowloon) than those who work in a low-risk district (e.g., New Territories). The potential mechanism underlying this result is the neighborhood effect averaging problem (NEAP) in people’s exposure to COVID-19 risk [21], which suggests that people who have the option to decide which trips to make or to forego can limit their exposure to high-risk places (e.g., working from home or conducting their daily activities in low-risk places). For instance, people who live in a low-risk neighborhood (e.g., Tin Shui Wai) may still have a high perceived COVID-19 risk if they have to work in a high-risk occupational venue.

Third, our results report that people’s age, marital status, educational level, income status, and housing conditions have strong associations with their perceived COVID-19 risk and distress. Specifically, people who are married and rent a residential unit have a higher level of neighborhood-based perceived COVID-19 risk and severe worries about job loss and reduced income compared to those who are single and own a residential unit or house. Meanwhile, a large household size (i.e., a high number of household members) would further worsen people’s worry about family conflicts. These results highlight the importance of housing conditions (e.g., homeownership and household size) in people’s distress during the pandemic. In addition, we also find that females have greater concern about job loss than males, while younger people have severe worry about family conflict compared to those who are older than 25 years of age. It should be noted that we do not find that people who live in *tong lau* and subdivided units have a significant increase in their perceived COVID-19 risk (see Table 7), although *tong lau* and subdivided units in Hong Kong have one of the highest infection rates in reported outbreaks internationally [23]. This result implies that people who live in *tong lau* and subdivided units might underestimate their risk of exposure to COVID-19 during the pandemic. The potential explanations include that people may have a false sense of a lower perceived COVID-19 risk under the zero-COVID strategy in Hong Kong, which allows people to enjoy long periods largely unencumbered by the pandemic [64,65].

Lastly, our findings show that people’s exposure to woodland during their daily activities significantly decreases their mobility-based perceived COVID-19 risk and distress. Meanwhile, the results do not report a significant association between people’s exposure to open space and recreational land and grassland with their distress (see Table 8). It should be noted that the woodland in Hong Kong is mainly distributed in country parks, and previous studies also found that people in Hong Kong prefer to visit country parks for their well-being during the COVID-19 pandemic [24,25]. However, these results are inconsistent with findings from previous studies [26,28,66], which concluded that people’s exposure to nearby community parks and private gardens is associated with better psychological well-being in Portugal, Spain, and the U.K. The differences in these results might be due to the urban built environments in the study areas: Hong Kong is a high-density city with an urban environment characterized by compactivity and connectivity [67], whereas Portugal, Spain, and the U.K. usually have cities with an urban environment characterized by dispersion and lower densities [68]. Thus, people in Hong Kong may have a higher willingness to go hiking in country parks that are located far away from their homes rather than taking a walk in nearby small community parks because of the social distancing orders during the pandemic.

In addition, our findings have several important implications for public health during the COVID-19 pandemic. First, our results suggest that people may be doubly disadvantaged due to their residential neighborhoods and poor housing conditions. In addition, people may underestimate their risk of exposure to COVID-19 even if they live in a high-risk neighborhood (e.g., Sham Shui Po) with poor housing conditions (e.g., *tong lau* and subdivided units). It is worth noting that previous studies have also indicated that people were disadvantaged in poor neighborhoods in different cities around the world (e.g., the U.K., U.S., and Brazil) during the pandemic [63,69,70]. Our study further provides empirical evidence from Hong Kong which emphasizes the critical challenges of poor neighborhoods in high-density cities in preventing the transmission of the pandemic. Specifically, our findings imply that the health authority and the government should put more resources (e.g., testing, vaccination, and financial support) to target certain vulnerable groups in the neighborhoods with poor housing conditions during the pandemic. In addition, the government should be aware that people might experience a heavy burden due to COVID-19 exposure risk and the related distress due to their poor housing conditions in a high-risk neighborhood during the current and future pandemics.

Second, our results confirmed the benefits of people’s daily exposure to greenspace (e.g., woodland) in decreasing their mobility-based perceived COVID-19 risk and distress during the pandemic. Considering the diverse benefits of greenspace exposure, the study recommends keeping the parks (e.g., country parks and nearby community parks) open and encouraging people to visit them during the pandemic. For instance, our study highlights the importance of people’s ability in their decision about daily mobility during the pandemic. Hence, policymakers should seek to improve people’s options for their daily mobility (e.g., improve their accessibility to parks and woodland).

Although the study is important because it highlights the importance of people’s residential neighborhoods, housing conditions, and daily greenspace exposure in their perceived COVID-19 risk and distress, it has some limitations that need to be addressed in the future. First, due to data limitations, the study only recruited participants aged 18–65 years old in two neighborhoods, which may not represent the vulnerable groups in other neighborhoods due to specific housing conditions (e.g., nursing homes) [34,71]. Future studies could further investigate how other types of housing conditions and greenspace exposure influence people’s perceived COVID-19 risk and distress for other groups (e.g., older adults) in various neighborhoods.

Second, there is much potential to further extend our study to other countries, regions, or cities. For instance, a comparative analysis of the associations between housing conditions and daily greenspace exposure with people’s perceived COVID-19 risk and distress in different cities (e.g., Tokyo, Beijing, Seoul, New York, London, and so on) could be conducted if similar surveys are conducted in these cities. Thus, future studies would reveal more systematical linkages between people’s housing conditions and daily greenspace exposure with their COVID-19-related worries. Moreover, our findings hint at disparities in both people’s perceived COVID-19 risk and distress from different residential neighborhoods, underscoring the need for future studies to examine whether and how health inequities have been exacerbated by COVID-19.

Lastly, our regression results also suggested that the models have relatively low Nagelkerke R^2^ scores (i.e., 0.115 to 0.191), which indicate that the independent variables (i.e., social-demographic features, housing conditions, and green space exposure) could explain 11.5% to 19.1% of the variability of the dependent variables (i.e., perceived COVID-19 risk and distress). One of the potential reasons might be the small sample size in this study. Future studies would benefit from using a large sample size based on similar survey methods to obtain more robust models.

## 5. Conclusions

Our study of people from two typical neighborhoods in Hong Kong highlights the importance of housing conditions and daily greenspace exposure for understanding the situations of socially vulnerable groups during the pandemic. People who live in high-risk neighborhoods and have poor housing conditions struggled with high neighborhood-based perceived COVID-19 risk and severe distress during the pandemic. Meanwhile, people also reported lower mobility-based perceived COVID-19 risk when compared to their neighborhood-based perceived COVID-19 risk during the pandemic, while they still have a high perceived COVID-19 risk in their occupational venues if they have to work in a high-risk district. In addition, daily greenspace exposure (e.g., woodland) reduces people’s perceived COVID-19 risk and distress, especially among those who have a big family in high-risk neighborhoods (i.e., married and have a large household size), and feel severe worries about job loss, income reduction, and family conflict. These results support the public health authority to target specific vulnerable social groups for supporting measures during the COVID-19 pandemic.

## Figures and Tables

**Table 1:** Sociodemographic characteristics of Sham Shui Po (*n* = 107) and Tin Shui Wai (*n* = 112) survey participants, and comparison with those of the neighborhood populations.

	Sham Shui Po (SSP)		Tin Shui Wai (TSW)	
Demographic Characteristic	Sample (*n* = 107)	Census Statistics	Sample (*n* = 112)	Census Statistics
Age Group				
18–24	16%	14%	21%	16%
25–44	46%	42%	48%	39%
45–64	38%	44%	31%	46%
Gender				
Male	44%	46%	47%	47%
Female	56%	54%	53%	53%
Monthly household income level (HKD)				
Less than 20,000	45%	55%	29%	45%
20,000–39,999	32%	27%	44%	34%
40,000 or over	23%	18%	27%	21%
Employment Status				
Housewife	12%	11%	14%	15%
Employed	80%	75%	73%	78%
Student	8%	14%	12%	7%

**Table 2:** Questions and items about people’s perceived COVID-19 risk and distress.

Questions about People’s Perceived COVID-19 Risk and Distress	Items
How severe do you think was the transmission of COVID-19 in your residential neighborhood from January 2020?	Neighborhood-based perceived COVID-19 risk
How severe do you think was the transmission of COVID-19 in venues or places you usually visited in one week?	Mobility-based perceived COVID-19 risk
Over the past year, how has your life been affected by COVID-19 pandemic?	Worry about job loss
	Worry about income reduction
	Worry about family conflict

**Table 3:** Descriptions of the social-demographic features, housing conditions, and greenspace exposure.

Variables	Description
Social-demographic features	
Residential neighborhood	Participants live in Sham Shui Po: 1; participants live in Tin Shui Wai: 0.
Gender	Participants are female: 1; participants are male: 0.
Age group 1	Participants are 18–24 years old: 1; otherwise: 0.
Age group 2	Participants are 45+ years old: 1; otherwise: 0.
Educational status	Participants have higher education degree: 1; otherwise: 0.
Marital status	Participants were married: 1; single, widowed, or divorced: 0.
Working place 1	Participants work in Hong Kong Island: 1; otherwise: 0.
Working place 2	Participants work in Kowloon: 1; otherwise: 0.
Income 1	Participants’ monthly household income < HKD 20,000: 1; otherwise: 0.
Income 2	Participants’ monthly household income > HKD 40,000: 1; otherwise: 0.
Full-time employed	Participants are full-time employed: 1; otherwise: 0.
Student	Participants are a student: 1; otherwise: 0.
Housewife	Participants are housewives: 1; otherwise: 0.
Housing conditions	
Homeownership (Rented)	Participants rent a residential house: 1; Participants own a residential house: 0.
Household size	The number of household members in participants’ residential units.
House type 1	Participants live in a public house: 1; otherwise: 0.
House type 2	Participants live in a *tong lau* or subdivided units: 1; Otherwise: 0.
Monthly household rent/mortgage payment 1	Participants pay HKD 1–4000 for the monthly rent/loan: 1; otherwise: 0.
Monthly household rent/mortgage payment 2	Participants pay HKD 4000–10,000 for the monthly rent/loan: 1; otherwise 0.
Monthly household rent/mortgage payment 3	Participants pay > HKD 10,000 for the monthly rent/loan: 1; otherwise: 0.
Green space exposure	
Open Space and Recreational land	The open space and recreation land around participants’ home/activity locations.
Woodland	The woodland land around participants’ home/activity locations.
Shrubland	The shrubland land around participants’ home/activity locations.
Grassland	The grassland land around participants’ home/activity locations.

**Table 4:** Descriptive statistics of people’s perceived risk and distress during the COVID-19 pandemic in the two neighborhoods: Sham Shui Po (*n* = 107), and Tin Shui Wai (*n* = 112).

	Sham Shui Po (SSP)	Tin Shui Wai (TSW)
People’s perceived COVID-19 risk		
Neighborhood-based risk	3.37 (0.96)	2.95 (0.77)
Mobility-based risk	2.48 (0.89)	2.51 (0.90)
Mean of difference ^a^	0.89 ***	0.42 ***
People’s distress		
Worry about job loss	3.39 (1.48)	2.58 (1.39)
Worry about income reduction	3.67 (1.49)	2.95 (1.42)
Worry about family conflict	3.23 (1.30)	2.83 (1.33)

Notes: Standard deviations in parentheses; ^a^ Paired sample *t*-test; *** denotes *p* < 0.001.

**Table 5:** Mann–Whitney U test results for the difference in people’s perceived risk and distress during the COVID-19 pandemic in the two neighborhoods: Sham Shui Po (*n* = 107), and Tin Shui Wai (*n* = 112).

	*p*-Value	∣r∣
People’s perceived COVID-19 risk		
Neighborhood-based risk	0.000 ***	0.22
Mobility-based risk	0.520	0.04
People’s distress		
Worry about losing job	0.000 ***	0.26
Worry about reducing income	0.000 ***	0.23
Worry about increasing family conflicts	0.042 *	0.13

Notes: r denotes effect size. *** denotes *p* < 0.001. * denotes *p* < 0.05.

**Table 6:** Rate of people’s high perceived risk and severe distress during the COVID-19 pandemic in the two neighborhoods: Sham Shui Po (*n* = 107), and Tin Shui Wai (*n* = 112).

	Sham Shui Po (SSP)	Tin Shui Wai (TSW)
People’s perceived COVID-19 risk		
Rate of high neighborhood-based risk	43%	19%
Rate of high mobility-based risk	10%	11%
People’s distress		
Rate of severe worry about job loss	53%	29%
Rate of severe worry about income reduction	59%	38%
Rate of severe worry about family conflict	45%	38%

**Table 7:** Results of the ordinal logistic regression models for people’s neighborhood-based risk (Model 1) and mobility-based risk (Model 2), in Sham Shui Po and Tin Shui Wai (*n* = 219).

Variables		Model 1 ^a^		Model 2 ^b^	
		Coef.	Std.	Coef.	Std.
Social-demographic features					
Residential neighborhood	Sham Shui Po	1.735 ***	0.797	−0.222	0.494
Gender	Female	−0.088	0.308	−0.052	0.308
Age	Age group 1 (18–24)	−0.313	0.421	−0.354	0.417
	Age group 2 (44–65)	0.035	0.399	0.373	0.404
Educational status	Higher education	0.787 *	0.409	0.758 *	0.422
Marital Status	Married	0.761 **	0.390	0.097	0.373
Working place	Hong Kong Island	−0.339	0.451	0.33	0.463
	Kowloon	0.265	0.356	0.791 ***	0.366
Monthly household income (HKD)	Income 1 (<20,000)	−0.466	0.357	−0.206	0.357
	Income 2 (>40,000)	−0.508 *	0.358	−0.316	0.36
Employment Status	Employed (full-time)	−0.206	0.421	0.402	0.395
	Student	0.705	0.570	0.816	0.561
	Housewife	−0.913	0.599	−0.237	0.602
Housing conditions					
Homeownership (Rented)		0.675 *	0.376	0.136	0.362
Household size		0.113	0.159	−0.01	0.157
House type	Public house	−0.750	0.508	−0.211	0.465
	*Tong lau* and subdivided units	0.728	0.571	0.142	0.545
Monthly household rent/mortgage payment (HKD)	Rent/mortgage payment 1 (1–4000)	0.619 *	0.375	0.383	0.362
	Rent/mortgage payment 2 (4000–10,000)	0.508	0.475	0.315	0.452
	Rent/mortgage payment 3 (>10,000)	0.106	0.518	−0.089	0.49
Greenspace exposure					
Open Space and Recreational land		−0.227	0.165	0.033	0.195
Woodland		0.169	0.230	−0.476 **	0.227
Shrubland		−0.299	0.286	−0.764 *	0.403
Grassland		−0.060	0.235	0.285	0.250
Intercept		−2.773 **	1.073	−1.221 ***	0.801
AIC		575.921		596.178	
Nagelkerke R^2^		0.191		0.115	

Notes: *** denotes *p* < 0.001. ** denotes *p* < 0.01. * denotes *p* < 0.05. ^a^ Dependent variable: the neighborhood-based risk; ^b^ Dependent variable: the mobility-based risk.

**Table 8:** Results of the ordinal logistic regression models for people’s worries about job loss (Model 3), income reduction (Model 4), and family conflict (Model 5) in Sham Shui Po and Tin Shui Wai (*n* = 219).

Variables		Model 3 ^a^		Model 4 ^b^		Model 5 ^c^	
		Coef.	Std.	Coef.	Std.	Coef.	Std.
Social-demographic features							
Residential neighborhood	Sham Shui Po	1.854 ***	0.476	2.287 ***	0.485	1.475 ***	0.478
Gender	Female	0.725 **	0.295	0.011	0.290	0.289	0.293
Age	Age group 1 (18–24)	−0.400	0.418	−0.266	0.422	0.695 *	0.421
	Age group 2 (44–65)	0.517	0.391	0.041	0.381	0.296	0.381
Educational status	Higher education	0.302	0.423	−0.182	0.410	−0.328	0.403
Marital Status	Married	0.880 **	0.354	0.704 **	0.347	0.464 *	0.360
Working place	Hong Kong Island	−0.526	0.448	−0.077	0.450	−0.095	0.447
	Kowloon	0.182	0.346	0.084	0.343	0.318	0.349
Monthly household income (HKD)	Income 1 (<20,000)	0.114	0.336	0.084	0.327	−0.209	0.333
	Income 2 (>40,000)	−0.263	0.350	−0.249	0.348	−0.383	0.349
Employment Status	Employed (full-time)	−0.074	0.409	−0.393	0.396	0.117	0.393
	Student	−0.537	0.578	−0.224	0.566	−0.239	0.582
	Household wife	−0.798	0.609	−0.695	0.587	−0.417	0.575
Housing conditions							
Homeownership (Rented)		0.520 *	0.358	0.619 *	0.354	0.283	0.341
Household size		0.266 *	0.148	0.286 *	0.147	0.512 ***	0.152
House type	Public housing	−0.091	0.464	−0.045	0.463	0.393	0.458
	*Tong lau* and subdivided units	0.586	0.546	0.817	0.557	0.646	0.573
Monthly household rent/mortgage payment (HKD)	Rent/mortgage payment 1 (1–4000)	0.291	0.357	0.070	0.346	0.248	0.352
	Rent/mortgage payment 2 (4000–10,000)	0.034	0.451	0.187	0.443	0.172	0.439
	Rent/mortgage payment 3 (>10,000)	0.083	0.474	−0.006	0.464	−0.428	0.465
Green space exposure							
Open Space and Recreational land		−0.138	0.179	−0.234	0.177	0.034	0.179
Woodland		−0.573 *	0.225	−0.517 *	0.219	−0.722 ***	0.216
Shrubland		0.443	0.417	−0.060	0.401	0.587	0.384
Grassland		0.088	0.211	−0.016	0.216	0.035	0.225
Intercept		2.546 ***	0.834	1.681 ***	0.810	1.964 ***	0.826
AIC		739.067		761.211		730.571	
Nagelkerke R^2^		0.143		0.129		0.121	

Notes: *** denotes *p* < 0.001. ** denotes *p* < 0.01. * denotes *p* < 0.05. ^a^ Dependent variable: score of worry about losing job; ^b^ Dependent variable: score of worry about reducing income; ^c^ Dependent variable: Score of worry about increasing family conflicts.

## Data Availability

Data sharing is not applicable to this article.
